# The Altemeier Procedure in the Management of Rectal Prolapse Secondary to a Prolapsed Rectal Polyp: A Valid Alternative

**DOI:** 10.7759/cureus.96216

**Published:** 2025-11-06

**Authors:** Andrés David De León Murillo, Pedro Antonio Plaza Ricardo, Tatiana Paola Pérez García, Juan Manuel Castro Rodriguez, Silvia Fernanda Anaya Meza, Ezio Pezzano Molina

**Affiliations:** 1 General Surgery, Universidad Libre, Barranquilla, COL; 2 General Surgery, Clínica Keralty, Ibagué, COL; 3 General Surgery, Hospital General de Barranquilla, Barranquilla, COL

**Keywords:** altemeier procedure, colorectal surgery, giant adenomatous polyp, rectal neoplasms, rectal prolapse

## Abstract

The definition of rectal prolapse is the protrusion of the rectal wall through the anal canal. The diagnosis of this condition is primarily clinical, based on the patient's symptoms and physical examination findings. Most rectal prolapses can be reduced spontaneously, or the patient can manually reduce them. On rare occasions, the prolapsed segment cannot be reduced, leading to secondary complications, such as severe pain, bleeding, strangulation, and even perforation. Its management is primarily surgical, and the choice of technique varies according to the severity of the condition, the symptoms presented, available resources, and the preferences of the patient and surgeon.

We present a case of a 67-year-old female patient with a history of a rectal polyp, who sought consultation for rectal bleeding and rectal prolapse. A giant rectal polyp was documented as the main cause of the prolapse, which was managed surgically with a perineal rectosigmoidectomy (Altemeier procedure). The pathological finding confirmed a giant adenomatous polyp with no evidence of malignancy. The patient had a favorable postoperative evolution.

## Introduction

Rectal prolapse is defined as the protrusion of the rectal wall through the anal canal. The following three types are recognized: full-thickness rectal prolapse, in which all layers of the rectal wall invaginate; mucosal prolapse, limited to the mucosa; and internal rectal prolapse, characterized by the invagination of the rectum into the anal canal without extending beyond the anal verge [1].

The incidence of rectal prolapse in adults ranges from 2.5 to 4.2 per 100,000 people, with a prevalence close to 1% in those over 65 years of age [1]. It is six to 10 times more common in women, especially those over 50 years of age and in multiparous women [1,2]. Although it can occur at any age, it is most prevalent in childhood and the geriatric population [1].

The pathophysiology of rectal prolapse is complex and multifactorial. The most commonly associated risk factors include multiparity, a history of vaginal delivery or pelvic surgery, pelvic floor dysfunction, chronic constipation or diarrhea, neurological diseases (such as dementia or cerebrovascular occlusion), and pelvic anatomical abnormalities (rectocele, cystocele, enterocele, among others) [1,2]. This condition significantly impacts quality of life and can manifest spontaneously or after maneuvers that increase intra-abdominal pressure, such as defecation or coughing [2].

The diagnosis of rectal prolapse is primarily clinical, based on the history and physical examination [3]. Complementary studies, such as colonoscopy, ultrasound, or magnetic resonance imaging, can rule out associated pathologies or predisposing conditions [1]. Although some cases can be reduced spontaneously or with manual maneuvers, the irreducibility of the prolapsed segment can lead to severe complications such as pain, bleeding, strangulation, or perforation [2].

Medical treatment plays a limited role, with surgery being the cornerstone of management [1]. Surgical techniques are grouped into abdominal and perineal approaches. Within the former, fixation and resection techniques offer lower recurrence rates but carry higher postoperative morbidity. The perineal approach, reserved for elderly patients or those with comorbidities, is associated with lower morbidity but a higher recurrence rate. Among these techniques, the most notable is perineal rectosigmoidectomy or Altemeier procedure, described for the treatment of full-thickness rectal prolapse [1].

On the other hand, rectal polyps are abnormal mucosal growths that are usually benign. Prolapsed mucosal polyps are lesions that can mimic inflammatory polyps and be associated with conditions such as solitary rectal ulcer syndrome or rectal prolapse [4,5]. Villous adenomas represent approximately 10% of colorectal adenomas and, although uncommon, have a risk of malignancy of up to 40% [6].

Rectal polyps rarely reach a size that allows protrusion through the anal canal, making rectal prolapse secondary to a giant adenomatous polyp an exceptional presentation [3]. In this context, the following case describes a giant tubulovillous adenoma that caused complete rectal prolapse and discusses the indication and results of the Altemeier procedure as a successful therapeutic approach in this unusual presentation.

## Case presentation

We present a case of a 67-year-old woman who presented to the emergency department with a week-long sensation of a mass protruding through the anus, accompanied by moderate rectal bleeding and persistent tenesmus. The patient reported a history of mild chronic constipation for several years, without associated fecal incontinence or abdominal pain. She reported no weight loss or recent changes in her bowel habits. Her past medical history included controlled high blood pressure and previous gynecological surgery (total abdominal hysterectomy).

Three months prior to admission, the patient underwent a diagnostic colonoscopy, which revealed a giant rectal polyp in the lower rectum. Histopathological examination of the endoscopic biopsy revealed a tubulovillous adenoma without high-grade dysplasia. Endoscopic resection was not performed due to the polyp's large size and broad base.

Upon initial physical examination, the patient was hemodynamically stable (blood pressure: 128/78 mmHg, heart rate: 84 beats per minute, temperature: 36.8°C) with no signs of respiratory distress or abdominal pain. Anal inspection revealed a full-thickness rectal prolapse with an edematous, friable polypoid mass measuring approximately 10 cm, with mild active bleeding that did not reduce spontaneously.

**Figure 1:**
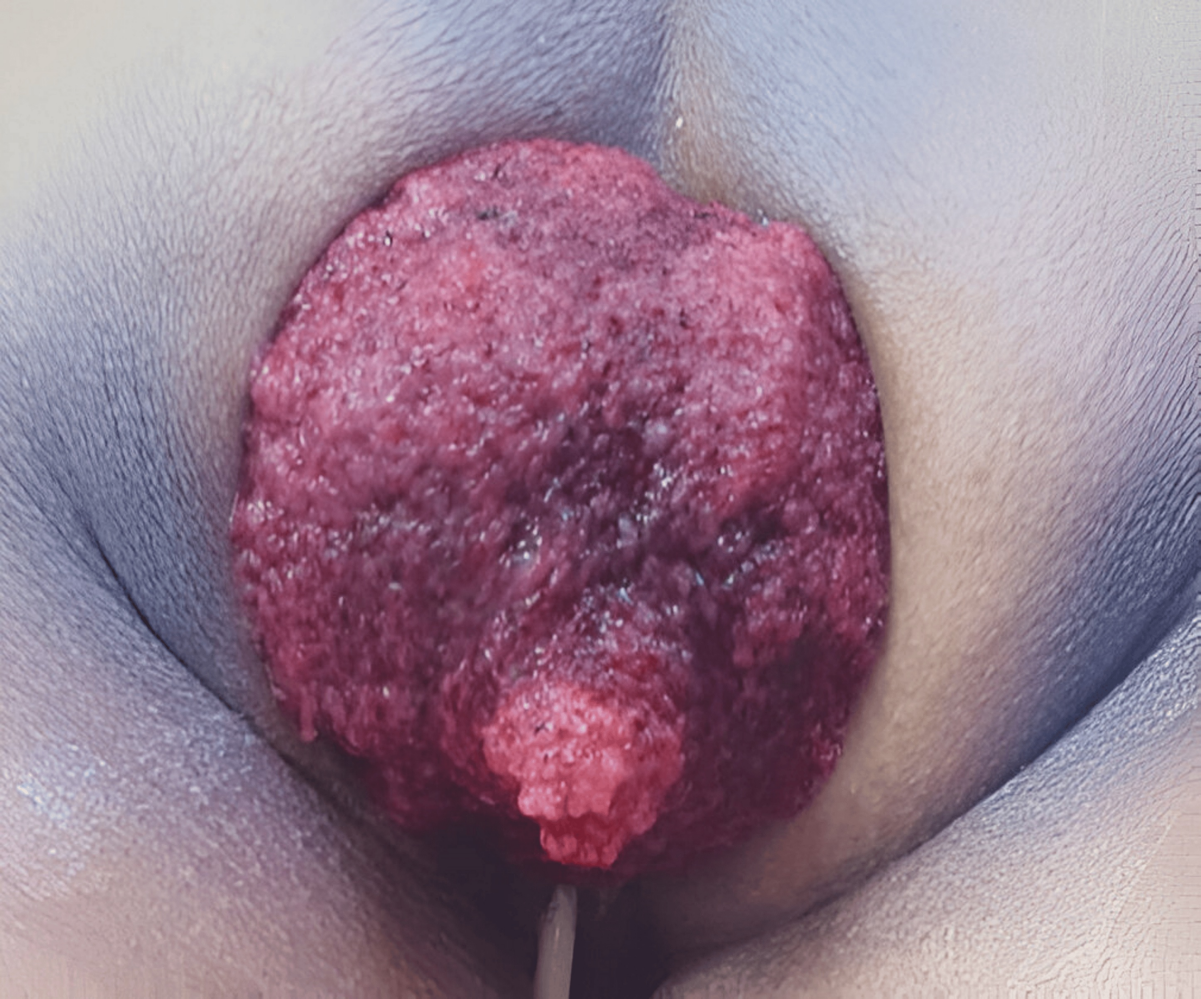
Patient in the prone position (decubitus prono) for the perineal approach to the Altemeier procedure. This position provides optimal exposure of the perineum and anal canal.

Initial conservative management was achieved with analgesia, prophylactic antibiotics, and preoperative bowel preparation. Given the persistence of the prolapse and the risk of necrosis of the prolapsed segment, a perineal rectosigmoidectomy (Altemeier procedure) was performed (Figure 1).

**Figure 2:**
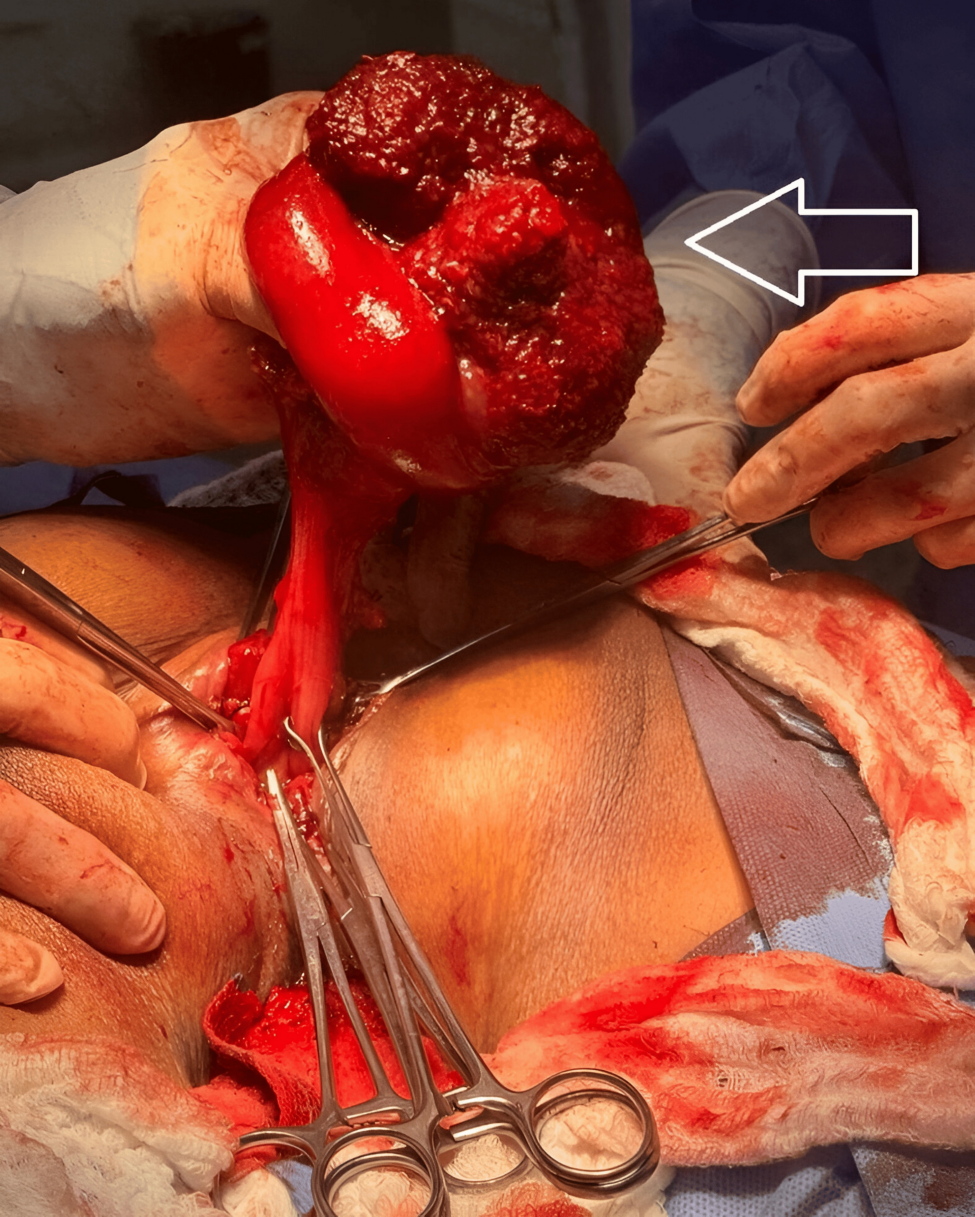
Intraoperative view of the prolapsed rectal segment. The white arrow indicates the large, pedunculated polyp originating from the proximal rectum, which was the starting point of the prolapse.

**Figure 3:**
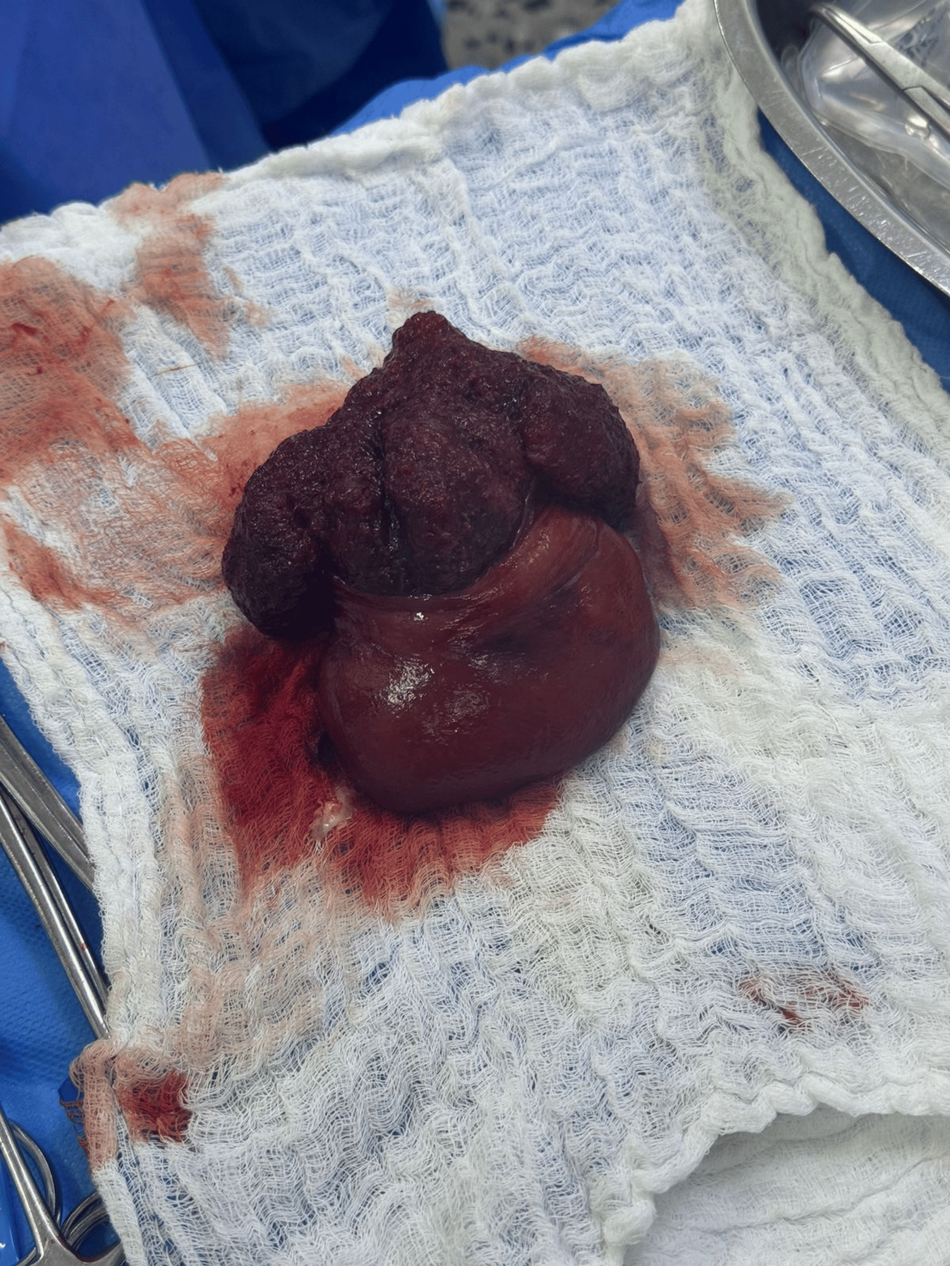
The surgical specimen after perineal rectosigmoidectomy (Altemeier procedure). The specimen includes the prolapsed rectal segment with a giant polyp measuring approximately 12x12 cm.

During surgery, a giant polyp measuring approximately 12×12 cm was identified in the proximal rectum, originating from the anterior wall and acting as a traction point responsible for the rectal prolapse (Figure 2). Complete resection of the involved rectosigmoid segment and manual end-to-end coloanal anastomosis were performed, with adequate perfusion and without tension. The surgical specimen was sent for pathological analysis (Figure 3).

**Figure 4:**
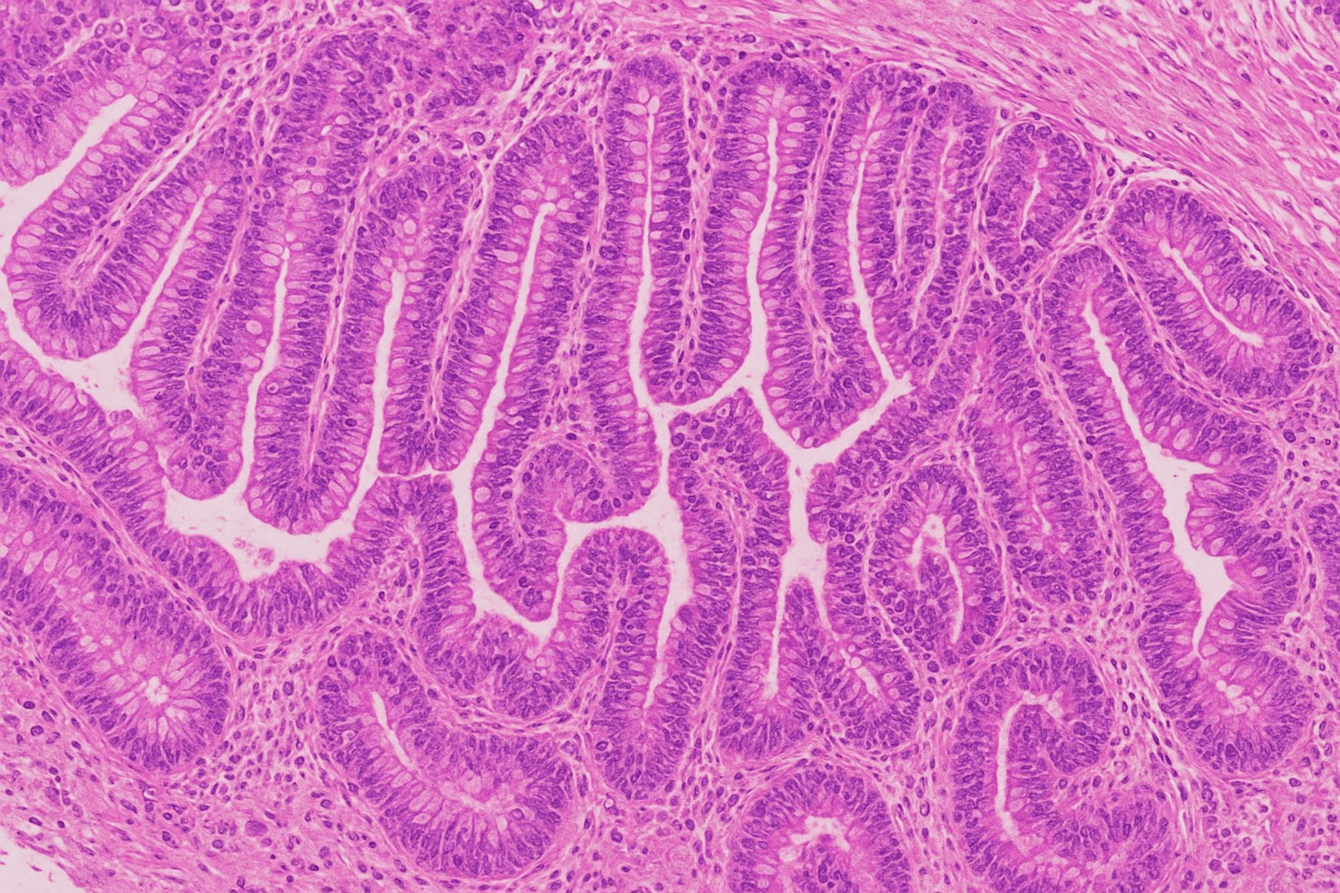
Photomicrograph of the polyp (H&E stain). Histology reveals a tubulovillous adenoma with low-grade dysplasia, characterized by a mixed tubular (60%) and villous (40%) pattern. No evidence of high-grade dysplasia or invasive carcinoma was observed.

Histological examination revealed the proliferation of glands lined by dysplastic epithelium showing a mixed tubular and villous pattern - predominantly tubular (60%) and villous (40%) - and low-grade dysplasia characterized by uniform nuclei and absence of atypical mitoses (Figure 4). No areas of carcinoma in situ or infiltration of the lamina propria were observed.

**Figure 5:**
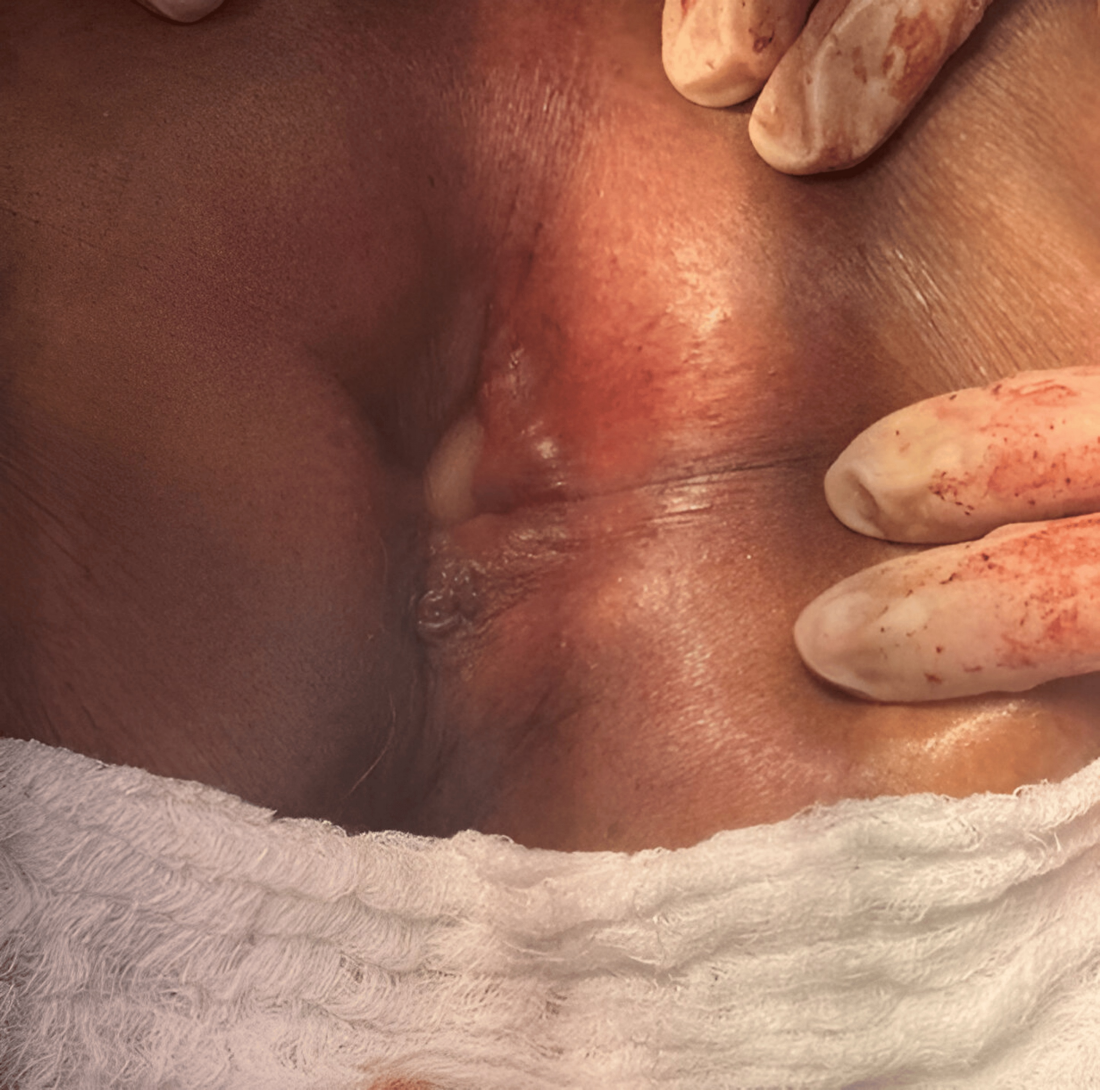
Final intraoperative view after completion of the coloanal anastomosis. The anastomosis is healthy and well-positioned within the anal canal after resection and extraction.

In the immediate postoperative period, the patient progressed favorably, with early restoration of intestinal transit, adequate oral tolerance, and no infectious or hemorrhagic complications. She was discharged on the fifth postoperative day with outpatient management and follow-up in the outpatient clinic. At the two-month evaluation, complete resolution of the rectal prolapse, adequate anal continence, and absence of recurrence or bleeding were evident (Figure 5).

## Discussion

Rectal prolapse associated with large polyps is a rare clinical entity that can pose a significant diagnostic and therapeutic challenge. The reviewed literature shows that although rectal polyps are common lesions in clinical practice, their presentation with prolapse, especially in adults, is unusual and may be associated with different etiologies, such as giant villous adenomas or the hamartomatous polyps characteristic of Peutz-Jeghers syndrome (PJS) [6-8].

Large villous adenomas, as described in the case by Munteanu et al., usually develop insidiously and reach significant sizes before producing symptoms. These may include rectal bleeding, prolapse, pain, and severe anemic syndrome. Their proximity to the dentate line and their circumferential nature make both diagnosis and endoscopic resection difficult, increasing the risk of coexisting invasive adenocarcinoma. This type of lesion requires careful surgical planning, as conservative techniques might be insufficient from an oncological point of view [6].

In the context of PJS, the presence of giant hamartomatous polyps with prolapse is extremely rare in adults. Cano-Contreras et al. reported a unique case in which an adult patient with a recent diagnosis of PJS presented with acute anal pain, bleeding, and prolapse of a necrotic polyp. Endoscopic resection was successful, but close follow-up is imperative in this type of patient due to the high risk of malignant transformation of these lesions over time [7].

On the other hand, the surgical review by Bordeianou et al. provides a broad conceptual framework for surgical decision-making in patients with rectal prolapse. Emphasis is placed on the need to individualize the approach according to the patient's age, functional symptoms (incontinence or constipation), pelvic anatomy, and comorbidities [8-12].

Several techniques for the management of rectal prolapse have been described, which are generally classified according to their approach, whether abdominal or perineal [13]. Abdominal techniques, such as ventral or posterior rectopexy with or without resection, posterior rectopexy with the use of mesh, Ripstein procedure, and laparoscopic rectopexy, offer better functional outcomes but require good functional reserve; however, they are associated with a higher mortality risk and may be associated with complications, such as impotence and infertility [8-13]. On the other hand, the perineal approach is associated with high recurrence rates; however, in frail or elderly patients, perineal approaches (Altemeier or Delorme) are appropriate and have acceptable recurrence rates when well-indicated [8-13].

Surgical intervention is more limited in cases of incarceration, due to the increased risk of anastomotic complications due to edema [13]. In cases where there is incarceration or ischemia, resection may be required, which is achieved through the perineal approach [13]. The objective of the procedure is to resect the affected and redundant intestine to prevent recurrence of the pathology [13].

The two techniques that are preferred for the management of strangulated prolapse vary depending on several factors. In elderly patients or those with multiple pathologies, the Delorme and Altemeier procedures are preferred, considering their lower postoperative complications, whereas in young and healthy patients, an abdominal approach is preferred [13]. Furthermore, it is emphasized that the perineal approach avoids the use of mesh, which may reduce the risk of infection at the new site [13].

Complications are reported in up to 10-12% of patients undergoing perineal rectosigmoidectomy, which indicates a low postoperative morbidity [13]. Among the postoperative complications, anastomotic leak stands out, which occurs in 2-6% of elective cases and up to 25% of cases in which there is incarceration [13]. The incidence of this complication is reported to be up to 10-15% in high-risk areas such as the rectum and approximately 5-10% in other locations [13]. Another critical factor is fecal continence; various studies report continence after the Altemeier procedure ranging from 21 to 100%, a percentage that varies depending on several factors, one that stands out is whether levatorplasty is performed [13]. Regarding the recurrence of rectal prolapse after the Altemeier procedure, a variability ranging from 6 to 40% is reported in the literature, which is also influenced by the performance of levatorplasty; however, some studies do not find a statistically significant difference in recurrence, whether levatorplasty was performed [13]. Regarding bowel habit after the Altemeier procedure, constipation is reported, although it is not very frequent [13]. Mortality, on the other hand, in the Altemeier procedure, is reported to be between 2% and 5% [13].

Recurrence is higher with perineal procedures than with abdominal procedures [13]. When comparing the Altemeier and Delorme perineal procedures, a variable postoperative recurrence of rectal prolapse has been described, 10-15% in patients who underwent the Altemeier procedure and 16-30% in patients who underwent the Delorme procedure, with statistically significant differences between the two procedures being observed in most studies [14].

The literature suggests that the ideal procedure in cases of incarceration is the Altemeier procedure, which is preferably performed in patients with comorbidities because it can be performed with regional or spinal anesthesia, avoiding the need for laparotomy and allowing for a faster recovery and early mobilization, also taking into account that it has a lower postoperative recurrence of rectal prolapse when compared to the Delorme procedure [13].

## Conclusions

Rectal prolapse secondary to large benign polyps is an infrequent but relevant condition, as it can be confused with other anal masses and, in certain cases, requires urgent surgical treatment. Giant villous adenomas present a high risk of malignant transformation, which necessitates a more radical approach when high-grade dysplasia or focal invasion is present.

In the present case, the Altemeier procedure proved to be a safe and effective management for rectal prolapse secondary to a giant, benign tubulovillous adenoma, resulting in complete symptom resolution. This supports its role as a valid perineal approach for elderly patients with this condition. More broadly, the literature review suggests that when managing rectal prolapse secondary to polyps, surgeons must consider the high malignant potential of some lesions, such as villous adenomas. In such cases, a more radical approach may be necessary. Furthermore, in syndromic patients (e.g., Peutz-Jeghers syndrome), polypoid prolapse is a recognized complication. Ultimately, surgical planning must be individualized based on patient factors and pathological risk.
